# Immunogenic Cell Death in Cancer Therapy

**DOI:** 10.32607/actanaturae.11523

**Published:** 2022

**Authors:** O. S. Troitskaya, D. D. Novak, V. A. Richter, O. A. Koval

**Affiliations:** Institute of Chemical Biology and Fundamental Medicine, Siberian Branch of the Russian Academy of Sciences, Novosibirsk, 630090 Russia; Novosibirsk State University, Novosibirsk, 630090 Russia

**Keywords:** Immunogenic cell death (ICD), HMGB1, calreticulin, antitumor vaccination, chemotherapy, apoptosis-inducing proteins, oncolytic viruses, cold plasma jet

## Abstract

Apoptosis plays a crucial role in chemotherapy-induced cell death. The conventional theory holding that apoptosis needs to be immunologically silent has recently been revised, and the concept of immunogenic cell death (ICD) has been proposed. This review describes the main features of ICD induction. These ICD markers are important for the effectiveness of anticancer therapy, as well as for basic research into cell death regulation. The mechanism of the "vaccination effect" of dying cancer cells undergoing ICD has been fully described, including the activation of specific antitumor response after re-challenge by the same living tumor cells. This review also discusses the whole set of molecular events attributing cell death to immunogenic type: the exposure of calreticulin and the heat shock protein HSP70 to the outer surface of the cell membrane and the release of the nuclear protein HMGB1 and ATP into the extracellular space. ICD inducers of various nature (chemotherapy drugs, cytotoxic proteins, and oncolytic viruses), as well as physical methods, are classified in the current review.

## INTRODUCTION

The long-held theory that tumor cells can be successfully eliminated only when they die via apoptosis, without activation of the immune system, has recently been revised. The "dual-action strategy" is one of the successful antitumor approaches outside of surgical intervention. In this strategy, on the one hand, an antitumor drug directly induces the death of most cancer cells, while, on the other, the dying cells activate the immune system and elicit a specific immune response to the tumor antigens, resulting in the destruction of the remaining tumor cells. These criteria are met by immunogenic cell death (ICD) inducers (this class includes antitumor drugs) and approaches that involve various mechanisms of action: conventional chemotherapeutics, protein-based drugs, oncolytic viruses, photodynamic and radiation therapies, as well as cold atmospheric plasma. Immunogenic cell death can be detected based on the activation of a certain combination of damage-associated molecular patterns (DAMPs) from dying tumor cells, which contributes to their recognition and uptake by antigen-presenting cells. The exposure of calreticulin and the heat shock protein HSP70 on the outer surface of the cell membrane, as well as the release of the nuclear protein HMGB1 and ATP into the extracellular space, is considered the key molecular event that allows one to talk about ICD induction [1, 2]. Tumor antigen processing and presentation by dendritic cells trigger the activation of antigen-specific T lymphocytes, thus eliciting an adaptive immune response against these antigens [3]. The activation of immunogenic cell death of tumor cells contributes to the eliciting of an adaptive immune response. Cells on the ICD pathway exhibit an anticancer vaccination effect when transplanted to syngeneic immunocompetent animals [4]. The development of a specific immune response against the antigens released by the dying tumor cells enables the use of therapeutic ICD inducers, both to assume control over metastatic tumors and to elaborate approaches to antitumor immunization [5].

## THE GENERAL CONCEPT OF IMMUNOGENIC CELL DEATH

The concept of tumor immunotherapy relies on the immune system’s ability to recognize transformed cells and affect their growth and proliferation. Physiological cell death occurs via apoptosis, which can be induced either by the organism’s intrinsic growth and life-sustaining programs by exposure to external factors [6]. Chromatin condensation, nucleus fragmentation with the plasma membrane remaining intact, and the emergence of apoptotic bodies are the morphological markers of apoptotic cell death, while plasma membrane integrity is disrupted during necrosis, resulting in the release of DAMPs activating the immune system and triggering an inflammatory response [7]. The proteins HMGB1, MRP8, calgranulins A and B, and MRP14 are the best studied DAMPs.

The differences in the antitumor properties of oxaliplatin and doxorubicin observed in experiments on immunodeficient and immunocompetent tumor-bearing mice have inspired scientists to search for an explanation to the phenomenon. Scheffer et al. [8] have put forward a hypothesis that when animals are subjected to antitumor vaccination with dying tumor cells, the repertoires of antigens from dying and intact cells may differ. Immunocompetent mice were transplanted with tumor cells: in some of those, apoptosis was induced by γ-irradiation, while in others necrosis was induced by freeze/thaw cycles. It was shown that when living tumor cells had subsequently been transplanted to the same mice, only animals vaccinated with apoptotic cells did not develop tumors in 75–100% of cases. Meanwhile, transplantation of living tumor cells did not result in tumor development in only 0–30% of animals vaccinated with necrotic cells on the same protocol. An immunohistochemical analysis of the vaccination site showed that the area had been infiltrated by CD4^+^ and CD8^+^ T cells and dendritic cells after the injection of apoptotic cells, which was an indication of a strong T-cell response, while the necrotic cell vaccine caused infiltration predominantly by macrophages [8]. Therefore, cells in which apoptosis was induced by γ-irradiation were found to exhibit an immunogenic potential. Tumor cells in which apoptosis was induced by anthracycline derivatives (e.g., doxorubicin) transplanted to mice were shown to stimulate the maturation of dendritic cells and subsequently elicit an immune response against tumor cells in vivo [4]. It was revealed by a comparison of the antitumor effects of treating immunocompetent and immunodeficient tumor-bearing mice with oxaliplatin or cardiac glycosides that the elimination of tumor cells occurs in immunocompetent mice, thus proving the role played by the immune system in the antitumor effects of these drugs [9, 10]. The apoptosis which causes the aforementioned effects is known as immunogenic apoptosis. A search for the molecular markers of immunogenic apoptosis showed that it is typically characterized by the secretion of DAMPs recognized by dendritic cells, followed by processing and presentation of antigens from the dying cells. This results in the activation of specific T cells and formation of long-lasting antitumor immunity [5].

## THE MECHANISM OF IMMUNOGENIC CELL DEATH INDUCTION

**The role played by the endoplasmic reticulum in ICD induction**

Doxorubicin, mitoxantrone, and γ-irradiation were the first efficient inducers of immunogenic cell death to appear on the scene. The ability of these antitumor drugs to trigger ICD was found to depend on their ability to induce endoplasmic reticulum (ER) stress [11]. The exposure of ER chaperones, primarily calreticulin (CRT), to the outer plasma membrane is the fundamental event in immunogenic cell death induction. When exposed to certain stimuli, the cell can trigger an integrated stress response, a complex molecular mechanism aiming to preserve cellular homeostasis [12]. In particular, anthracycline-induced ER stress stimulates PERK, which phosphorylates the translation initiation factor eIF2α [13]. Inactivation of eIF2α is accompanied by partial activation of caspase 8 and cleavage of B-cell receptor-associated protein 31 (BAP31) and conformational activation of the Bax and Bak proteins; in turn, it triggers translocation of ER chaperones to the outer cell membrane [11]. For most ICD inducers, the translocation of chaperones to the outer membrane does not occur directly but results from their transport from ER to the Golgi apparatus, mediated by vesicle-associated membrane protein 1 (VAMP1) and synaptosomal-associated protein 25 (SNAP25), and requires concomitant production of reactive oxygen species (ROS) [11, 14, 15]. According to Garg et al. [16], if the ER-to-Golgi transport is blocked, the exposure to ICD inducers reduces the secretion of ATP into the extracellular space, while not causing CRT exposure, which suggests that calreticulin and ATP follow the ER-to-Golgi transport pathway to reach the plasma membrane. The ICD-induced translocation of CRT to the outer plasma membrane is apparently regulated by multiple factors: the CXCL8 chemokine ligand [17], the changes in the Ca^2+^ levels in the ER [18], caspase 2 [19], long non-coding RNAs (e.g., ncRNA-RB1 and miR-27a) [20], and plasma membrane integrins, at least under some conditions [21]. CRT and other ER chaperones on the cell surface contribute to the uptake of these dying cells or their fragments; they are referred to as "eat-me" signals for antigen-presenting cells (APCs) [16]. Furthermore, the exposure of CRT apparently stimulates type I IFN secretion by antigen-presenting cells [22], which may also contribute to the immunogenicity of regulated cell death.

It has been shown that simultaneous elevation in the cellular level of ROS and induction of ER stress activate the signal pathways that help transport DAMPs into the extracellular space [11, 23]. Interestingly, immunogenicity decreases in the presence of antioxidants, thus indicating that ROS are crucial for ICD induction [11, 24]. It was later found that cisplatin, which alters the cellular redox metabolism, cannot trigger ICD, because it is unable to induce ER stress [25]. Furthermore, the simultaneous ER stress and ROS production increases the amount of various, released DAMPs, which eventually becomes a crucial factor for the immunogenicity of dying tumor cells [16, 26]. Thus, etoposide causes only exposure of HSP70 and ATP secretion but neither induces ER stress nor triggers ICD [23, 27, 28].

**Classification of ICD**

Two types of ICD inducers are currently distinguished depending on whether they trigger apoptosis through ER, or apoptotic cell death and ER stress occur independently [29]. Such agents as doxorubicin or mitoxantrone can be classified as type I ICD inducers (i.e., agents that trigger apoptosis through non-ER targets and stimulate the ICD-associated immunogenicity through the secondary or "side" stress effects of the ER). Contrariwise, type II ICD inducers selectively target the ER components and can induce immunogenic apoptosis by directly altering the ER homeostasis and triggering ER stress (e.g., photodynamic therapy). Therefore, ER stress triggered by type I ICD inducers can differ qualitatively from that triggered by type II inducers, since it can be less severe and capable of initiating the transducing survival-promoting signals [29].

In addition to immunogenic apoptosis, other types of programmed cell death include autophagy, necroptosis, and pyroptosis involving activation of some ICD markers. Table 1 lists the variants of immunogenic cell death and their specific features.

**Table 1:** Comparison of different types of programmed cell death in cells manifesting immunogenicity

Type of cell death	DAMPs characteristic of ICD	“Eat-me” signals	Inflammation	Immunogenicity	Terminal cellular events
Apoptosis	Ecto-CRT, secretion of HMGB1 and ATP	Ecto-CRT, HSP70, HSP90, exposure of PS	-	+	Nonlytic pathway, DNA fragmentation and apoptotic bodies
Autophagy	Release of HMGB1 and ATP	Secretion of LPC, exposure of PS	-	+	Nonlytic pathway, autophagic bodies
Necroptosis	Long genomic DNA, IL-6 [30], ATP, and HMGB1 [31]	Secretion of LPC, exposure of PS, low level of ecto-CRT [31]	+	++	Nonlytic pathway, loss of plasma membrane integrity, swelling of cellular organelles
Pyroptosis	Release of HMGB1, ATP, IL-1α, IL-1β, IL-6, IL-18, and TNF-α	Exposure of PS	+	++	Lytic pathway, plasma membrane rupture, release of the cell contents

Note. The degree of immunogenicity for each type of cell death was assessed as + and ++ depending on the intensity of “eat-me” signals and the level of DAMP release [30].

**Immunogenic cell death cascade**

**Fig. 1:**
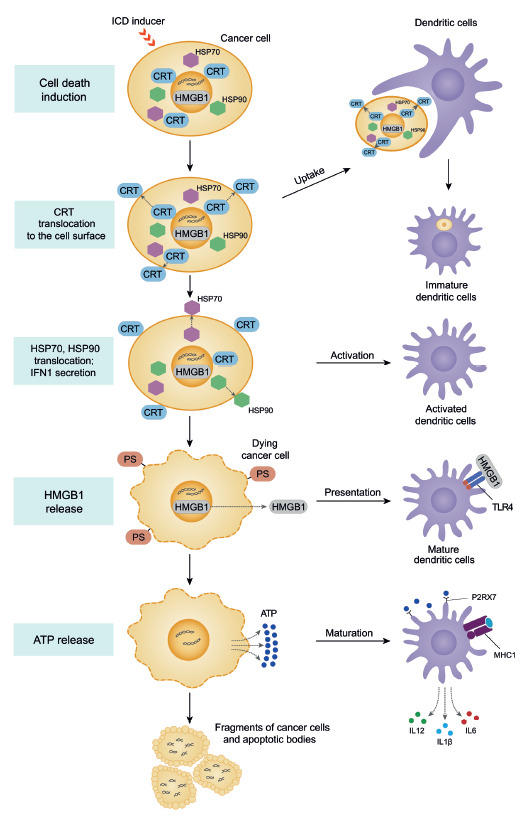
Sequential events of immunogenic cell death and activation of antigen-presenting dendritic cells

The key molecular events required for immunogenic cell death to take place have been identified (Fig. 1). The first event of the ICD cascade is the exposure of a complex formed by two proteins, calreticulin and disulfide isomerase ERp57, on the surface of dying tumor cells [11]. Both proteins are normally located in the ER lumen and are translocated to the cell surface within a few hours after stimulation with ICD inducers. CRT exposure can be detected before the translocation of phosphatidylserine (PS) to the outer membrane of a dying cell. CRT translocation from the ER is an initiating "eat-me" signal for phagocytic cells. Calreticulin exposed on the cell membrane interacts with the CD91 receptors on the surface of dendritic cells, thus stimulating the uptake of dying cells [29, 32].

Another molecular feature of ICD that can be observed after the CRT exposure consists in the translocation of heat shock proteins (such as HSP70 or HSP90, which can bind to the CD91 receptor on the dendritic cell surface like calreticulin) from the nucleus to the cell surface, which stimulates their activation and maturation [33].

Twelve to 18 hours after the initiation of CRT exposure, non-histone chromatin-binding nuclear protein HMGB1 is released into the intercellular space. This protein binds to the TLR4 receptors in dendritic cells, which is required to ensure optimal TLR4-dependent processing and presentation of tumor antigens to T cells by dendritic cells [34]. During chemotherapy or radiation therapy, dendritic cells receive a signal through TLR4 and its adapter, MyD88, to start efficient processing and cross-presentation of antigen from dying tumor cells [35]. The final molecular event in the ICD cascade is the release of ATP into the extracellular space, which is the "find-me" signal and is required for productive maturation of dendritic cells. The dying cells mark their presence through chemotactic factors known as "find-me" signals that are needed so that phagocytic cells (neutrophils, monocytes, and tissue macrophages) could quickly find and efficiently destroy them [36]. The release of ATP from dying cells into the intercellular space activates the P2X7 purinergic receptors on dendritic cells and causes P2X7/NLRP3 receptor-dependent activation of the inflammasome in dendritic cells, thus contributing to proteolytic maturation and the release of proinflammatory cytokines such as interleukin IL-1β. IL-1β is essential for the activation of antigen-specific CD8^+^ T cells producing IFNγ [3]. Moreover, IL-1β is involved in the activation of the innate immunity factors, development of inflammation, and the early stages of the immune response [34, 37].

**Fig. 2:**
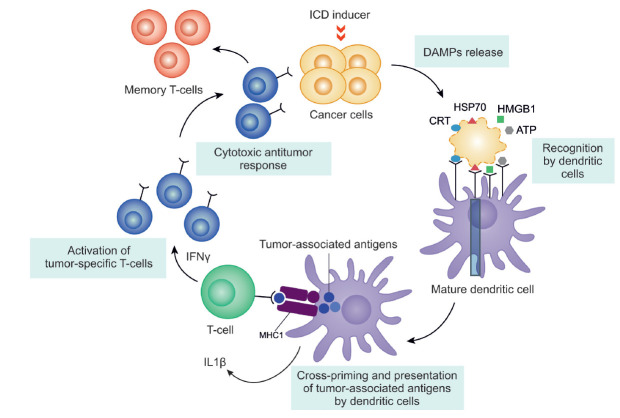
A simplified scheme of the induction of immunogenic cell death

If the cascade of immunogenic apoptosis is successful, a population of antigen-specific T cells is expected to emerge: when being re-challenged with tumor cells of this type, antigen-specific T cells will recognize the respective antigens and destroy cancer cells (Fig. 2). The possibility of inducing the cascade of events for immunogenic apoptosis in tumor cells using antitumor drugs has enabled us to develop an antitumor vaccination strategy where cells with induced immunogenic cell death are the "vaccine."

## THE ENDOGENIC FACTORS INVOLVED IN IMMUNOGENIC CELL DEATH

**Calreticulin (CRT)**

Approximately 30% of all cell proteins and peptides are synthesized in the ER, where they interact with enzymes and chaperons, including calreticulin, calnexin, glucose-regulated protein Grp94, thiol oxidoreductases PDI, and protein disulfide isomerase ERp57. All these molecules are involved in the formation of the functional conformation of proteins [38]. CRT, calnexin, and ERp57 constitute the chaperone complex responsible for the folding of the synthesized proteins transported through the ER and their quality control.

Another important function of the ER is storing and releasing Ca^2+^ ions [39]. Calreticulin, a unique Ca^2+^-binding chaperone, is one of these proteins [40]. Cells with downregulated CRT expression are characterized by protein misfolding and accumulation of misfolded proteins [40]. Overexpression of CRT increases the Ca^2+^ content in intracellular depots [41].

**Fig. 3:**
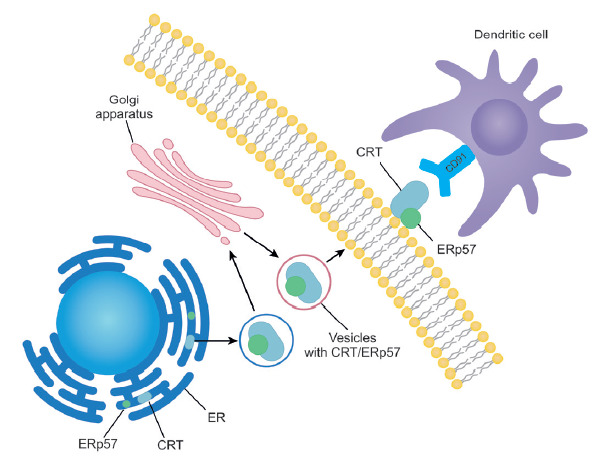
The exposure of calreticulin (CRT) on the cell surface and its recognition by dendritic cells

It is assumed that the cell surface CRT plays a role in antigen presentation, activation of the complement system [42], apoptotic cell removal [43], immunogenicity of dying cancer cells [23], wound healing [44], and thrombospondin signaling [45]. CRT acts as a secondary ligand on the cell surface, being essential for recognition during phagocytosis and stimulating LRPs (low-density lipoprotein receptor-bound proteins) on the surface of engulfing cells. The protein resides on the outer surface of the plasma membrane in many cell types, where it may contribute to antigen processing and mediate cell–cell adhesion [40]. Being normally located in the lumen of the endoplasmic reticulum, CRT is translocated to the outer cell membrane in the form of a complex with ERp57 as a result of ER stress via exocytosis (Fig. 3). The ER-to-membrane transport of CRT depends on the interaction between vesicle-associated SNARE (V-soluble N-ethylmaleimide-sensitive factor attachment protein receptor) proteins and the SNARE proteins on the cell membrane [11, 21]. Calreticulin on the outer plasma can bind to the CD91 receptors in dendritic cells, thus causing phagocytosis of dying cells [46].

**The signaling function of ATP in the activation of the immune system**

Dying cells mark their presence by releasing chemotactic factors (known as "find-me" signals) and through the "eat-me" signals that act as ligands for uptake. Several factors that can act as "find-me" signals have been proposed, including ATP, UTP, the chemokine fractalkine (CX3CL1), lysophosphatidylcholine (LPC), and S1P [47]. Apoptotic cells are converted to secondary necrotic cells when their scavenging is disrupted, which causes chronic inflammation and the development of autoimmune diseases [35].

The release of ATP into the extracellular space is typical of both immunogenic apoptosis and necro sis, accompanied by cell lysis. However, there exist several differences between these processes. The first difference is related to the amount of released ATP. During apoptosis, less than 2% of cellular ATP reaches the extracellular space [48]. The characterization of ATP as a mediator of inflammation largely rests on its ability to activate the ionotropic nucleotide receptor P2X7, which, in turn, causes the activation of the inflammasome and release of proinflammatory cytokines [49]. The enormous release of ATP during necrosis activates the inflammasome and the inflammation process. Nonetheless, the ATP concentration required to activate purinergic P2X7 receptors is no less than 100 μM, significantly higher than that required to activate chemotactic receptors such as P2Y2 ( < 1 μM) [50]. Interestingly, lower ATP concentrations can actually exhibit an anti-inflammatory effect by inhibiting the secretion of inflammatory cytokines, as well as promoting the release of anti-inflammatory cytokines [35]. Hence, ATP cannot be regarded as a universal signal of inflammation development.

**The non-histone chromatin-associated nuclear protein HMGB1 and its functions in the cell**

The HMGB1 protein belongs to the HMG (High mobility group) family: the family of nuclear non-histone proteins required to maintain chromatin architecture. Inside the cell, HMGB1 interacts with p53, TBR, Oct14, Hox, steroid hormone receptors, and many viral proteins and efficiently regulates gene expression [51]. HMGB1 can migrate between the cytoplasm and the cell nucleus depending on the phase of the cell cycle. Lymphoid cells contain HMGB1 both in the cytoplasm and in the nucleus [52].

The emergence of HMGB1 in the intercellular space is considered a marker of sudden damage or necrosis, since chromatin is damaged irreversibly in this case. In the mechanical damage foci, HMGB1 interacts with the receptor for advanced glycation end products (RAGE), thus enhancing the production of TNF, IL-1, IL-8, MCP1, CDF1α, and other factors, recruiting healthy stem cells to the damage focus [53]. HMGB1 can be secreted in cells both actively and passively. The active secretion of HMGB1 is related to the dissociation from the complex with chromosome damage resulting from histone acetylation, HMGB1 hyperacetylation, and monomethylation of HMGB1. Passive diffusion of HMGB1 is observed during necrosis. However, in the case of normal (non-immunogenic) apoptosis, HMGB1 is not released from the tightly packed apoptotic cell nuclei [54]. According to Luo et al. [54], the release of HMGB1 from necrotic tumor cells treated with doxorubicin, which causes necrosis when used at high concentrations [55], contributes to the resumption of tumor growth and metastasis development via the RAGE system activation pathway.

**Heat shock proteins HSP70 and HSP90**

Transcription activation of a number of chaperones belonging to the class of inducible HPS proteins or heat shock proteins is a common response to cellular stress, including stress induced by chemotherapeutics. Heat shock proteins protect the cell against death by refolding the damaged proteins or directing the damaged proteins to proteasomes for degradation [34].

In mammals, HSP70 is involved in protein formation, stabilization, and transport across the mitochondrial and nuclear envelopes [56]. Chaperone HSP90 performs a number of functions in the cell, including protein folding and stabilization under heat shock; it also promotes protein degradation [57]. Chaperone HSP90 stabilizes many of the proteins that are responsible for tumor growth and is involved in the regulation of adhesion, invasion, metastasis, angiogenesis, and apoptosis; therefore, HSP90 inhibitors are studied as potential antitumor agents [58].

Furthermore, the heat shock proteins HSP70 and HSP90 can form complexes with peptide antigens, including tumor-targeting peptides, which is a necessary and sufficient source of antigens for presentation to T cells. Unbound peptide antigens cannot elicit the T-cell response in CD8^+^ lymphocytes, unlike the antigens bound to heat shock proteins. In vivo experiments conducted on mice have demonstrated that the complexes formed between antigens, on the one hand, and HSP70 and HSP90, on the other, can be a source of antigens for efficient cross-presentation by dendritic cells [59].

## IN VIVO INDUCTION OF IMMUNOGENIC CELL DEATH UPON PROPHYLACTIC VACCINATION

**Fig. 4:**
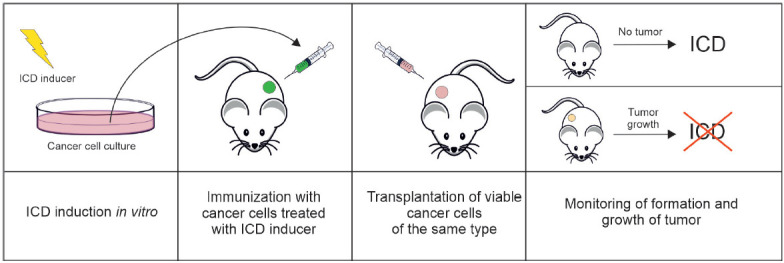
The classical scheme of antitumor vaccination with mouse tumor cells treated with a potential ICD inducer, followed by re-vaccination with viable tumor cells of the same type

Today, there exist several models for in vivo ICD studies. The "gold standard" for evaluating the ability of dying cells to trigger adaptive immunity involves prophylactic vaccination of immunocompetent syngeneic animals [5]. In this approach, tumor cells are exposed in vitro to a potential ICD inducer and then transplanted subcutaneously as a vaccine containing no immunological adjuvants. One to two weeks later, the animals are re-challenged with viable tumor cells of the same type at the minimum dose required for the formation of tumor nodules; tumor growth is monitored for 40–60 days (Fig. 4) [4, 35, 60]. Not only is the percentage of tumor-free mice taken into account for assessing the vaccination effectiveness, but allowance is also usually made for the tumor growth rate if tumors develop regardless of the vaccine-induced adaptive immunity. The specificity of the development of an antitumor response is confirmed by the fact that at the end of the experiment, tumor-free vaccinated mice were re-challenged with syngeneic cancer cells of a different line, which are expected to cause neoplastic progression in 100% of mice. The potentiated effectiveness of therapy with any inducer of regulated death of tumors growing in immunocompetent mice compared to immunodeficient ones indicates that this inducer has the potential to trigger ICD. However, this experimental design does not allow one to distinguish between ICD induction and non-ICD immunostimulation. Some antitumor drugs (such as docetaxel, cisplatin, 5-fluorouracil, gemcitabine, etc.) do not induce ICD but mediate immunomodulatory effects in the tumor microenvironment by having a direct impact on immune cell populations. Although these immunomodulatory effects are crucial for maximizing the clinical effectiveness of therapy, they are not related to ICD induction [12, 61].

An equivalent approach to the in vivo evaluation of ICD in immunocompetent syngeneic systems can consist in measuring the growth of a tumor located far from the tumor structure treated using local ionizing radiation or intratumoral delivery of anticancer therapy [62]. This approach is also effective when the tumor is accessible only to cytotoxic T lymphocytes (CTLs) (e.g., in the case of brain metastases in a patient receiving chemotherapy agents that cannot cross the blood–brain barrier) [63]. The models of the so-called "latent response" (i.e., regression of tumor lesions located far away from the site of the ionizing radiation treatment of the primary tumor in patients) proved useful in this situation [64]. This ex vivo modeling of ICD induction allows one to characterize DAMPs released by tumor cells in response to in situ stress, perform immunological profiling of the APCs and CTLs that underlie the in vivo initiation and implementation of antitumor immunity, and identify the sequences of the triggered ICD cascades and their correspondence to the observed responses in vitro.

## DRUGS INDUCING IMMUNOGENIC CELL DEATH

**Chemotherapeutics**

Induction of immunogenic cell death was first demonstrated for doxorubicin, an anthracycline drug [4]. Some chemotherapeutic agents can also induce ICD (selected drugs are listed in Table 2) [9, 65, 66, 67].

**Table 2:** Chemotherapeutics inducing immunogenic apoptosis

Chemotherapeutics	Types of tumor cells	Markers of ICD induction, DAMPs	Vaccination effectiveness, %
Anthracyclines (doxorubicin, daunorubicin, and idarubicin), doxorubicin-loaded liposomes[4, 68]	Murine CT26 colon carcinoma	CRT exposure, ER stress, eIF2α phosphorylation, HMGB1 release, ATP secretion	Doxorubicin, 80; Daunorubicin, 35; Idarubicin, 45
Oxaliplatin [9, 69, 70, 71]	Murine CT26 colon carcinoma, RKO and HCT116 human colorectal carcinoma	CRT exposure, HMGB1 release	Oxaliplatin, 80
Microtubule inhibitors (colchicine, CMQ, FMQ, nocodazole, epothilone B, Taxotere)[67, 72]	Murine CT26 colon carcinoma	ER stress, CRT exposure, PERK-dependent phosphorylation of eIF2α	Nocodazole, 80
Cardiac glycosides (digoxin DIG, digitoxin DIGT) [65, 73]	MCA205 mouse fibrosarcoma, murine B16 melanoma	CRT exposure, HMGB1 release, ATP secretion	DIG/DIGT +; cisplatin – 70–90; DIG/DIGT +; mitomycin – 60–90

**Peptides exhibiting antitumor activity**

Peptide LTX-315. Some peptides exhibiting an antitumor activity can also induce ICD. Thus, such cationic amphiphilic synthetic peptide as LTX-315 permeabilizes the inner mitochondrial membrane and causes necrotic cell death [74]. Intratumoral injections of LTX-315 completely eliminate murine B16 melanoma, while mice treated with the drug exhibit resistance to subsequent injections of live B16 melanoma cells. Peptide LTX-315 activates all the key molecular markers of ICD: CRT exposure, release of HMGB1 and ATP, as well as interferon response without activation of cellular caspases, which suggests that cell death occurs via the non-apoptotic pathway [74, 75].

The antitumor peptide RT53 belonging to the CPP class. The synthetic antitumor peptide RT53 belonging to the CPP class (high-permeability proteins) causes tumor cell death through unregulated necrosis with markers of ICD [76]. It was shown that after vaccination with RT53-treated B16F10 melanoma cells, only 25% of mice had no tumors at the re-transplantation site [77]. The development of antitumor immunity induced by RT53 peptide was also confirmed in C57BL/6 mice prophylactically vaccinated with RT53-treated MCA205 mouse fibrosarcoma cells: only the tumor growth rate decreased, but tumors at the re-transplantation site were not completely eliminated [76].

RIG-1-like helicases. The group of peptide inducers of ICD also includes RIG-1-like helicases. In contrast to LTX-315 and RT53, the RIG-like helicase RIG1 triggered apoptosis of Panc02 mouse pancreatic tumor cells with markers of ICD. Along with the conventional set of ICD markers, increased production of interferons and some proinflammatory cytokines was observed. Importantly, dendritic cells in the spleen efficiently engulf tumor cells treated with RIG-1 and present tumor-associated antigens to naïve CD8^+^ T cells [78].

Recombinant analog of lactaptin (RL2). Recent studies have shown that a recombinant analog of the human milk pro-apoptotic protein lactaptin (RL2) [79, 80] can induce ICD in vitro by activating the whole cascade of immunogenic cell death markers and elicit an antitumor immune response in the prophylactic vaccination model [81]. Thus, in experiments on immunocompetent C3H/He mice, 43% of mice vaccinated with RL2-treated MX-7 murine rhabdomyosarcoma cells did not develop a tumor nodule after they had been re-challenged. It is also worth mentioning that the growth rate of tumors that had actually developed was significantly lower compared to the control group. Ethyl pyruvate, an indoleamine 2,3-dioxygenase inhibitor, used in combination with cells incubated in the presence of RL2 potentiated the vaccination effect of RL2-treated cells by up to 60% [81].

**Oncolytic viruses in ICD induction**

It has been demonstrated that the death of cells infected with some unmodified oncolytic viruses, such as the Newcastle disease virus, measles virus, vaccinia virus (VV), and coxsackievirus B3, occurs with the activation of typical ICD markers [82, 83, 84]. The abilities of the human adenovirus, Semliki Forest virus, and wild-type VV to induce ICD were compared. All three viruses were found to stimulate the release of ICD markers, as well as the activation and maturation of dendritic cells; however, only the tumor cells infected with the Semliki Forest virus stimulated T-helper type 1 (Th1) maturation and induced antigen-specific T-cell activation [85]. Dendritic cells phagocytizing tumor cells infected with VV were unable to elicit a T-cell response. On the other hand, attenuated VV strains activated the STING- and Batf3-dependent pathways in dendritic cells and induced potent antitumor immunity [86]. Therefore, modification of the VV genome can be considered as a strategy to overcome the immunosuppression characteristic of wild-type VV. Heinrich et al. [84] showed that when incubated with human melanoma cells, the JX-594 (Pexa-Vec) virus causes exposure of CRT, HMGB1 release, and dendritic cell activation/maturation. The VV-GMCSF-Lact recombinant virus causes the death of tumor cells of different histological origins with markers of ICD [87, 88]. It has been revealed recently that glioma therapy with the Newcastle disease virus elicits an adaptive immune response against glioma cells, being a component of the antitumor response [89]. The recombinant adenovirus carrying the CD40 ligand transgene induces a type 1 T-helper response, resulting in the activation of cytotoxic T cells and reducing immunosuppression [90].

**Physico-chemical approaches to antitumor therapy with an ICD-inducing potential**

It has been demonstrated that various approaches involving physical impact (e.g., ionizing radiation, photochemotherapy, photodynamic therapy, near-infrared photoimmunotherapy, high hydrostatic pressure, thermal shock, nano-pulsed stimulation, hyperthermia, and cold plasma irradiation) can induce the death of tumor cells with markers of ICD [12].

Radiation therapy. Radiation therapy is among the methods of local tumor treatment; however, ionizing radiation also causes the elimination of tumor cells in distant metastases, thus indicating that radiation activates the immune system [91]. In vitro experiments have shown that radiation therapy induces a dose-dependent death of triple-negative breast cancer cells with exposure of CRT and release of ATP and HMGB1 [92]. In order to potentiate the immunogenic component of radiotherapy, it is also used in combination with clinically effective chemotherapeutics, causing immunogenic cell death (e.g., oxaliplatin or paclitaxel) [92].

Hyperthermia. It has been shown that exposure to heat shock above 42°C (hyperthermia) can induce a cascade of events that trigger ICD in vitro and elicit immunogenicity in mice. Thus, prophylactic vaccination with CT26 tumor cells exposed to heat shock (47°C) significantly inhibits tumor growth in the site of living cells inoculation and increases the survival chances of vaccinated animals [93].

Nano-pulse stimulation. It has been shown that nano-pulse stimulation leads to complete regression of weakly immunogenic metastatic 4T1-Luc murine mammary carcinoma [94]. Another interesting observation is that spontaneous metastases to distant organs were detected less frequently even in animals in whom tumor had not regressed completely. After nano-pulse stimulation and tumor regression, all mice became resistant to re-challenging with tumor cells and exhibited a vaccination-like effect. Nano-pulse stimulation was shown to induce antitumor immunity, stimulate the maturation of memory T cells, cause the destruction of the tumor microenvironment, and reduce the number of immunosuppressive cells in the tumor microenvironment and blood.

Cold atmospheric plasma (CAP). Cold atmospheric plasma (CAP) is one of the novel, promising directions in the therapy of malignancies. Cold atmospheric plasma treatment leads to selective death of melanoma cells [95], intestinal [96] and lung cancer cells [97, 98], pancreatic [99], gastric [100] and breast cancer cells [101], as well as glioblastoma cells [102] in vitro.

Cold atmospheric plasma irradiation can also trigger immunogenic cell death. Death of Hmel1 MM melanoma cells and PANC-1 pancreatic tumor cells treated with a CAP-irradiated culture medium was shown to be accompanied by CRT exposure and ATP release, which suggests that plasma-activated media can potentially be used as an inducer of cell death through activation of innate immunity [103]. Even a CAP-irradiated phosphate buffer can trigger the ICD cascade in vitro [104]. Direct treatment of tumor cells with CAP can also trigger ICD by inducing the exposure of calreticulin and HSP70 on the outer membrane, as well as secretion of ATP and HMGB1 [105]. It was also found that in vitro CAP treatment of tumor cells causes the release of ICD-specific DAMPs; 30% of mice vaccinated with CAP-irradiated CT26 cells did not develop tumors at the site of re-challenging with live tumor cells, while 90% of the tumors that developed in vaccinated mice were smaller compared to the average tumor size in the control group [106]. In vivo cold plasma irradiation of MX-7 rhabdomyosarcoma tumors transiently increased the serum levels of HMGB1 in tumor-bearing animals [105].

Hence, some physical methods of cancer therapy can be regarded as ICD inducers and the contribution of the antitumor immune response to tumor therapy effectiveness in patients can be evaluated.

## SUPPRESSION OF THE ANTITUMOR IMMUNE RESPONSE UPON ICD INDUCTION

Along with the endogenous factors that activate the immune system, there are several mechanisms that serve to suppress the immune response through inhibitory signals. As a tumor progresses, it acquires a number of properties that allow it to evade the immune system [107]. The tumor microenvironment prevents the penetration of tumor infiltrating lymphocytes by limiting the nutrient supply and by releasing inhibitory signals. Plasmacytoid dendritic cells, tumor-associated macrophages and myeloid-derived suppressor cells secreting anti-inflammatory cytokines and expressing immunosuppressive metabolic enzymes (such as inducible nitric oxide synthase (iNOS), indoleamine 2,3-dioxygenase (IDO), tryptophan 2,3-dioxygenase (TDO), and arginase) play an important role in the development of the immunosuppressive tumor microenvironment [108, 109]. The reduction in the tryptophan level because of the action of IDO1 and the simultaneous increase in the level of its metabolites stimulate the immunosuppressive properties of the tumor and its microenvironment mainly through the development of APC- and T-cell-mediated immune tolerance, as well as immune cell death [110]. This suppression of the T-cell metabolism can inhibit the effector activity of T cells, while simultaneously stimulating regulatory T cells and acting as a barrier to effective immunotherapy. Rapid depletion of nutrients such as glucose and accumulation of metabolic products such as lactate or kynurenine, which directly inhibit T cells, are characteristic of tumors [111]. Along with signals such as CRT, which recruit cells that exhibit phagocytic activity, tumor cells can display molecules that are antagonistic to "eat-me" signals (CD47 molecules) on their surface, resulting in the suppression of calreticulin-mediated phagocytosis. The interaction between CD47 and the SIRPα receptor on dendritic cells is a signal that inhibit phagocytosis [112]. Activation of the aforementioned mechanisms can potentially interfere with the ICD cascade and protect tumor cells against attacks on the immune system.

## CONCLUSIONS

Immunogenic cell death is a unique response that is initiated by cellular stress and ends in cell death, accompanied by the active secretion or passive release of numerous alarmins. The ICD plays a crucial role in fighting a cancer thanks to its ability to trigger the antitumor immune response, potentiating the therapeutic effect of chemotherapeutics and radiation therapy agents. Detailed research into the molecular markers of ICD will allow us to better predict the in vivo activation of the antitumor immune response by using specific antitumor drugs and approaches.

## Abbreviations

APCs: antigen-presenting cells
ATP: adenosine triphosphate
CAP: cold atmospheric plasma
CRT: calreticulin
CTLs: cytotoxic T lymphocytes
DAMPs: danger-associated molecular patterns
ER: endoplasmic reticulum
HMG: high-mobility group
HSP: heat shock protein
ICD: immunogenic cell death
IL: interleukin
LPC: lysophosphatidylcholine
MHC: major histocompatibility complex
PS: phosphatidylserine
ROS: reactive oxygen species
TLR: Toll-like receptor
TNF: tumor necrosis factor
VV: vaccinia virus.
